# Chronische vernarbende Plaques: ein Fortschritt in der Behandlung

**DOI:** 10.1007/s00105-025-05603-z

**Published:** 2025-10-28

**Authors:** Maximilian Lammer, Paul Bellmann, Barbara C Böckle

**Affiliations:** Abteilung für Dermatologie und Venerologie, Medizinische Universität Innsbruck, Anichstr. 35, 6020 Innsbruck, Österreich

**Abb. 1:**
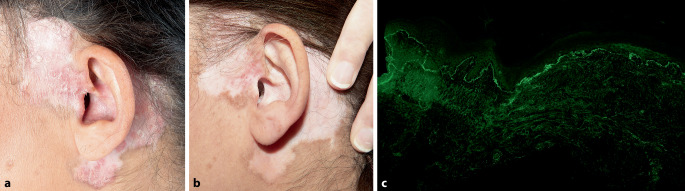
Runde, hypopigmentierte vernarbte Plaques mit erythematösem Randsaum im linken Schläfenbereich (**a**) mehrere Tage nach Erstgabe von Anifrolumab (**b**) Stanzbiopsie, direkte Immunfluoreszenz; granuläre Ablagerungen von Immunglobulin M und Komplement 3 entlang der Basalmembranzone (**c**)

50-jährige Patientin mit seit 9 Jahren bestehenden vernarbten Plaques im Gesicht (Abb. 1a).

Die Laborergebnisse einschließlich ANA, ANCA, C3, C4, HIV und Anti-dsDNA-Antikörper waren bis auf Lupus-Antikoagulans und verlängerte Lupus sensitive aPTT unauffällig. Diese zeigten sich nach 12 Wochen gemäß Sydney-Kriterien persistierend. Aufgrund eines Pons-Infarkts wurde ein Antiphospholipidsyndrom diagnostiziert. Bei der internistischen und neurologischen Untersuchung wurden keine weiteren Auffälligkeiten festgestellt.

Eine Stanzbiopsie aus dem linken Schläfenbereich zeigte eine atrophe Epidermis mit einer Interface-Dermatitis an der dermoepidermalen Junktionszone und einer perivaskulären und perifollikulären lymphozytären Infiltration. Die direkte Immunfluoreszenz zeigte granuläre Ablagerungen von Immunglobulin M und Komplement 3 entlang der Basalmembranzone (Abb. 1c).

Es wurde die Diagnose eines chronischen diskoiden Lupus erythematodes (CDLE) gestellt – EULAR/ACR-Kriterien waren nicht erfüllt.

Trotz mehrerer Behandlungen – lokale Glukokortikoide, lokale Calcineurininhibitoren, Hydroxychloroquin 200 mg 2‑mal täglich seit 7 Jahren, Methylprednisolon niedrig dosiert intermittierend, Mepacrin 100 mg 1‑mal täglich in Kombination mit Hydroxychloroquin für ein Jahr sowie Methotrexat 10 mg wöchentlich (Dosissteigerung aufgrund von Übelkeit nicht möglich) in Kombination mit Hydroxychloroquin für 3 Monate – wurde über Jahre hinweg kein klinisches Ansprechen beobachtet (Abb. 1a). Daher wurde nach ausführlicher Aufklärung und schriftlichem Einverständnis eine Off-label-Therapie mit Anifrolumab 300 mg, einem Antikörper gegen Typ-I-Interferonrezeptoren (IFNAR1), alle 4 Wochen begonnen. Einige Tage nach der ersten Verabreichung erstmals signifikante klinische Besserung (Abb. 1b).

Der CDLE ist die am häufigsten auftretende Form des kutanen Lupus erythematodes.

Die Erstlinientherapie besteht aus topischen Glukokortikoiden und dem Malariamittel Hydroxychloroquin. Allerdings sprechen < 50 % der Fälle darauf langfristig klinisch an, was oft eine Langzeitbehandlung mit oralen Kortikosteroiden erfordert [1]. Die Pathogenese ist multifaktoriell und umfasst genetische Veranlagung, UV-induzierte Apoptose und dysregulierte Immunreaktionen, bei denen Interferon‑α eine Schlüsselrolle spielt [2].

Unser Fall zeigt in Übereinstimmung mit der vorhandenen Literatur, dass die Behandlung mit Anifrolumab behandlungsresistenten CDLE klinisch signifikant verbessern kann [3].
